# Age-Dependent Changes in Bone Architecture, Patterning, and Biomechanics During Skeletal Regeneration

**DOI:** 10.3389/fcell.2021.749055

**Published:** 2021-10-13

**Authors:** Kevin Hoffseth, Emily Busse, Josue Jaramillo, Jennifer Simkin, Michelle Lacey, Mimi C. Sammarco

**Affiliations:** ^1^Department of Biological and Agricultural Engineering, Louisiana State University, Baton Rouge, LA, United States; ^2^Department of Surgery, Tulane School of Medicine, New Orleans, LA, United States; ^3^Department of Orthopaedic Surgery, Health Sciences Center, Louisiana State University, New Orleans, LA, United States; ^4^Department of Mathematics, Tulane University, New Orleans, LA, United States

**Keywords:** regeneration, bone, aging, biomechanics, elastic modulus, digit regeneration

## Abstract

Mouse digit amputation provides a useful model of bone growth after injury, in that the injury promotes intramembranous bone formation in an adult animal. The digit tip is composed of skin, nerves, blood vessels, bones, and tendons, all of which regenerate after digit tip amputation, making it a powerful model for multi-tissue regeneration. Bone integrity relies upon a balanced remodeling between bone resorption and formation, which, when disrupted, results in changes to bone architecture and biomechanics, particularly during aging. In this study, we used recently developed techniques to evaluate bone patterning differences between young and aged regenerated bone. This analysis suggests that aged mice have altered trabecular spacing and patterning and increased mineral density of the regenerated bone. To further characterize the biomechanics of regenerated bone, we measured elasticity using a micro-computed tomography image-processing method combined with nanoindentation. This analysis suggests that the regenerated bone demonstrates decreased elasticity compared with the uninjured bone, but there is no significant difference in elasticity between aged and young regenerated bone. These data highlight distinct architectural and biomechanical differences in regenerated bone in both young and aged mice and provide a new analysis tool for the digit amputation model to aid in evaluating the outcomes for potential therapeutic treatments to promote regeneration.

## Introduction

The ability to regenerate limb structures, where new growth replaces both the amputated bone and surrounding soft tissue, varies widely in vertebrates. While the axolotl is able to completely regenerate an entire limb after amputation, *de novo* regeneration is extremely limited in mammals. In rodents, monkeys, and humans, regeneration is restricted to only the distal one-third of the third phalangeal element (P3). More proximal amputations, either in the same bone, in more proximal bones (P2), or through long bones such as the femur, result in the formation of a hypertrophic callus and failed regeneration. The regenerative response of the digit tip has been well documented in rodents, monkeys, and humans (Bryant et al., 2002; Brockes and Kumar, 2005; Han et al., 2008; Fernando et al., 2011; Simkin et al., 2013); and significant efforts have been placed on dissecting out the distinguishing signaling pathways differentiating a regenerative vs. a non-regenerative amputation. However, little information exists regarding the biomechanical properties of the regenerated bone. This information gap is largely due to the unusual shape and size of P3, and the fidelity of regenerative outcomes has been based predominantly on the ability to recapitulate the bone architecture and not biomechanical properties of the regenerated bone, such as hardness and elasticity.

To overcome these limitations and explore the biomechanical properties of skeletal regeneration, we utilized both nanoindentation and micro-computed tomography (microCT) image-processing methods for the assessment of elasticity in the whole P3 bone (Hoffseth K. et al., 2021) and evaluated the impact of aging on bone elasticity after regeneration. In this study, we showed that the elasticity measurements calculated from our processing method are able to detect age-dependent differences in elasticity that small samples of Young’s modulus values, generated from directly measured reduced modulus values from nanoindentation, are not able to detect (Hoffseth K. et al., 2021).

By using this processing method, we found that age increases the elasticity of the distal tip of the unamputated digit, while the process of regeneration predictably decreases elasticity and hardness. Surprisingly, we found that both calculated elasticity and direct measurements of hardness show no significant difference between the 6-month-old (Hoffseth K. et al., 2021) and 18-month-old mice after regeneration. These biomechanical similarities are in contrast with age-dependent differences seen in the bone architecture of young and aged mice. Together these findings underscore the importance of age-dependent bone architecture and suggest that the elasticity of aged regenerated bone maintains high fidelity when compared with young bone. These findings are the first step toward addressing, at least in part, the biomechanical properties of regenerated bone, whether endogenous or engineered, and suggest that interventions to address differences in bone architecture would be impactful. Moving forward, approaches such as finite element modeling, which ties together architecture and biomechanics, will be immensely useful in predicting and evaluating the regenerative outcomes of potential treatment in this digit regeneration model.

## Materials and Methods

### Amputations and Animal Handling

Adult 18- to 20-month-old male and female CD1 wild-type mice were purchased from Charles River (Wilmington, MA, United States). Mice were anesthetized with 1–5% isoflurane gas with continuous inhalation. The second and fourth digits of both hind limbs were amputated at the P3 distal level as described previously (Fernando et al., 2011; Sammarco et al., 2014; Busse et al., 2019), and regenerating digits were collected at day 42 for analysis. The third digit was used as an unamputated control.

### Tissue Collection, Fluorescence, and Imaging

Digits were fixed overnight in zinc-buffered formalin (Z-fix; Anatech). Bone was decalcified for 48 h in a formic acid-based decalcifier (Decal I; Surgipath). Once decalcified, all samples were processed for paraffin embedding. Immunofluorescent staining was performed on deparaffinized and rehydrated sections. Antigen retrieval was performed using antigen retrieval solution (Vector, H-3300) prior to blocking with blocking solution (Thermo, 37515). Sections were incubated with anti-CD31 antibody (Abcam, ab182981) overnight at 4°C and subsequently incubated with fluorescently labeled secondary antibodies (1:500) for 1 h at room temperature. Slides were imaged using a Cytation5 with 492/520 nm filter set (*N* = 3, each group). Registration of the P3 microCT stack to the corresponding CD31 image was performed using FIJI (ImageJ). MicroCT stacks were imported into FIJI as an image sequence in 8-bit grayscale. An arbitrarily oriented cross-sectional slice of the digit was reconstructed from its corresponding set of microCT images, visualized through rotation of the slice plane in relation to the *xy*, *yz*, and *xz* planes using the Volume Viewer plugin (https://imagej.nih.gov/ij/plugins/volume-viewer.html), with tricubic interpolation of voxel values for rendering. The cross-sectional slice was visually compared with the digit CD31 slide, with the best-fit image found by iterative variation of slice rotation in *xy*, *yz*, and *xz* planes and manual matching to the bone in the CD31 slide. The final image was then created through an overlay of the best-fit image on the CD31 slide image.

### Micro-Computed Tomography and Density Calculations

*Ex vivo* microCT images of mouse digits were acquired using a Bruker SkySkan 1172 scanner (Bruker, Kontich, Belgium) at 50 kV and 201 μA, with 2K resolution and an isotropic voxel size of 3.9 μm. Images were captured at a rotation angle of 0.2 with a frame averaging of five. The complete P3 bone was used for analysis. Raw images were processed with Nrecon and Data Viewer (Bruker, Kontich, Belgium). For density calculations, attenuated x-ray data values were calibrated to mineral density using standard 0.25 and 0.75 mg hydroxyapatite density phantoms and converted to grayscale output. Hounsfield units (HU) values obtained from each phantom scan were used to calibrate for bone mineral density (BMD) within the CTan program. Density heatmaps were generated using Bruker software program CTvox where the colorized scale represents the corresponding tissue mineral density values throughout the rendered volume of each sample.

### Bone Architecture

Skeletonization and spatial BMD were performed as described previously (Hoffseth K. F. et al., 2021). A three-dimensional parallel thinning algorithm was applied to microCT image data processed by digit, operating on calculated internal void geometry. Skeleton analysis returned a total sum of all internal skeletonized segment lengths by digit. Skeletonization raw data were normalized to the average skeletonization value for unamputated digits from an 18-month-old mouse.

### Nanoindentation

Nanoindentation was performed by the Mayo Clinic Biomechanics Core (Rochester, MN, United States). Mouse P3 digits (*N* = 3 digits, each group) were stripped of all soft tissue and frozen before being embedded in polymethyl methacrylate in acrylic cylinders. Using a combination of a low-speed diamond saw and a polishing/grinding system, the digits were sectioned along the sagittal plane. Once the cross-section of a bone was revealed, it was manually polished using successively finer abrasive cloths (400, 600, 800, and 1,200 grit), with a final polish using a microcloth and slurry of 0.05-μm aluminum abrasive. Indentation testing was conducted on the cortical bone with a nanoindentation system (TI 950, Hysitron, Minneapolis, MN, United States) equipped with a diamond Berkovitch pyramidal tip. A total of 8 sites, 5 distributed across the distal area, and 3 distributed across the proximal area, were tested on each bone. At each site, a 2 × 2 array was indented with 15 μm spacing between indents. Indentation was conducted under load control at a rate of 500 μN/s to a peak load of 2,000 μN with a 60 s hold before unloading to reduce viscoelastic effects. The reduced modulus (*E*_r_; GPa) and hardness (*H*; GPa) were calculated using the Oliver–Pharr model (Oliver and Pharr, 1992, 2004).

### Density-Elastic Modulus Calculation

The reduced modulus measurements acquired from nanoindentation were used to calculate Young’s modulus using the Oliver–Pharr model (Oliver and Pharr, 1992, 2004):


$$\frac{1}{E_{r}}=\frac{1-\nu^{2}}{E}+\frac{1-\nu_{i}^{2}}{E_{i}}$$

where ν = 0.3 for bone, and *E*_*i*_ = 1,141GPa, ν_*i*_ = 0.07 for the diamond indenter, where ν = Poisson’s ratio of bone, *E*_*i*_ = Young’s modulus of the indenter material, and ν_*i*_ = Poisson’s ratio of the indenter.

Calculation of elastic modulus values utilized a processing pipeline starting with BMD as measured by μCT and using established density-elasticity relationships (Knowles et al., 2016) as detailed in Hoffseth K. et al. (2021) using 6-month-old mice. Mineral density values were calculated as previously described (Hoffseth K. F. et al., 2021) by averaging grayscale pixel intensity using L3-sized voxels (*L* = 3 pixels) for each representative data point through iterative operation over the digit image stack, reducing computation time without avoiding loss of digit characteristics. We utilized our predictive processing method (Hoffseth K. et al., 2021) to calculate elasticity values for the entire P3 bone in unamputated digits (all cortical bone) and day 42 regenerated digits (both cortical and trabecular bone). As previously described (Hoffseth K. et al., 2021), the density-modulus relationship equation below was used to calculate values of elasticity that were calculated from μCT measured volumetric BMD values using quantitative computed tomography (Knowles et al., 2016).


$$E=10,200\,\rho_{a\,s\,h}^{2.01}\;\text{with}\;\rho_{a\,s\,h}=0.8772\,\rho_{H\,A}+0.0789$$

### Statistical Analysis

Young’s modulus (*E*) and hardness (*H*; *N* = 3, age 6 months; *N* = 3, age 18 months; one digit per mouse) were analyzed using the two-way ANOVA models for the bone area (proximal or distal) and amputation status (UA or D42) with random effects at the level of mouse and nanoindentation site within mouse using the R package nlme (Pinheiro et al, 2021). Statistical significance was assessed by testing the set of relevant contrasts (UA proximal vs. distal, D42 proximal vs. distal, and UA distal vs. D42 distal) with adjustment for multiple comparisons using the R package multcomp (Hothorn et al., 2008). Numerically calculated elastic modulus values were reduced to 1,000 values per digit and location (proximal or distal) *via* random sampling. Samples were analyzed with the two-way ANOVA models for the bone area and amputation status with random effects at the mouse level using nlme and multcomp as described above. Local polynomial regression curves were fit to the full set of calculated elastic modulus values for each digit as a function of proximal–distal location using the R function “loess” with span parameter set to 0.33. Differences in the distribution of BMD (*N* = 5 digits, age 18 months; *N* = 6 digits, age 6 months) were analyzed using linear quantile mixed models using the R package “lqmm.” Models were fit to assess differences in the 10th, 25th, 75th, and 95th percentiles of the distributions of UA and D42 digits as a function of age, using samples of 2,000 density values per digit with random effects at the level of mouse. Using the same approach, models were also fit to assess differences between UA and D42 digits between 6-month-old and 18-month-old mice.

## Results

### Internal Void Skeletonization

**FIGURE 1:**
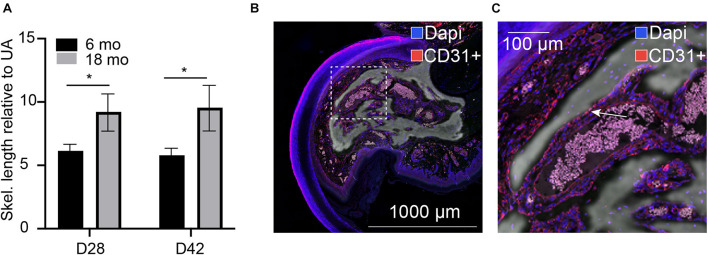
Internal void space. **(A)** Skeletonization of the internal vascular space of the regenerated digit at day 28 and day 42 relative to the unamputated skeletonization length in 6-month-old and 18-month-old mice. *indicates *p* < 0.05. *N* = 11–14 digits. Data are represented as mean ± SEM. **(B,C)** Void spaces are lined with CD31-positive cells. CD31 (red), dapi (blue). Inset shows **(C)** close-up CD31-positive area. Arrow indicates CD31^+^ cells. Representative sample shown: 18-month D42, *N* = 3. Scale bar = 1,000 μm.

We previously demonstrated that traditional quantification of bone morphometrics and analysis often does not appropriately address the long, highly variable, vascular-like spaces that are seen during digit skeletal regeneration. We used our previously described skeletonization technique (Hoffseth K. F. et al., 2021) to compare young and aged regenerated digits. For these comparative studies, we used 6-month-old mice, given that the growth ends and the skeleton stabilize around 6 months of age, following post-pubertal changes (Glatt et al., 2007). Using this analytical approach, we compared our previously analyzed 6-month-old digits (Hoffseth K. F. et al., 2021) with our aged digits and found that aged digits have significantly greater lengths of vascular space at day 28 when compared with young regenerated bone at day 28 and that this increase is sustained through day 42 (Figure 1A). Registration of CD31 staining for endothelial cells to the corresponding microCT in an aged digit shows that these inner void spaces are lined with CD31-positive cells (Figures 1B,C). These data support that bone morphology, vasculature, and architecture are impacted by age during skeletal regeneration.

### Density

**FIGURE 2:**
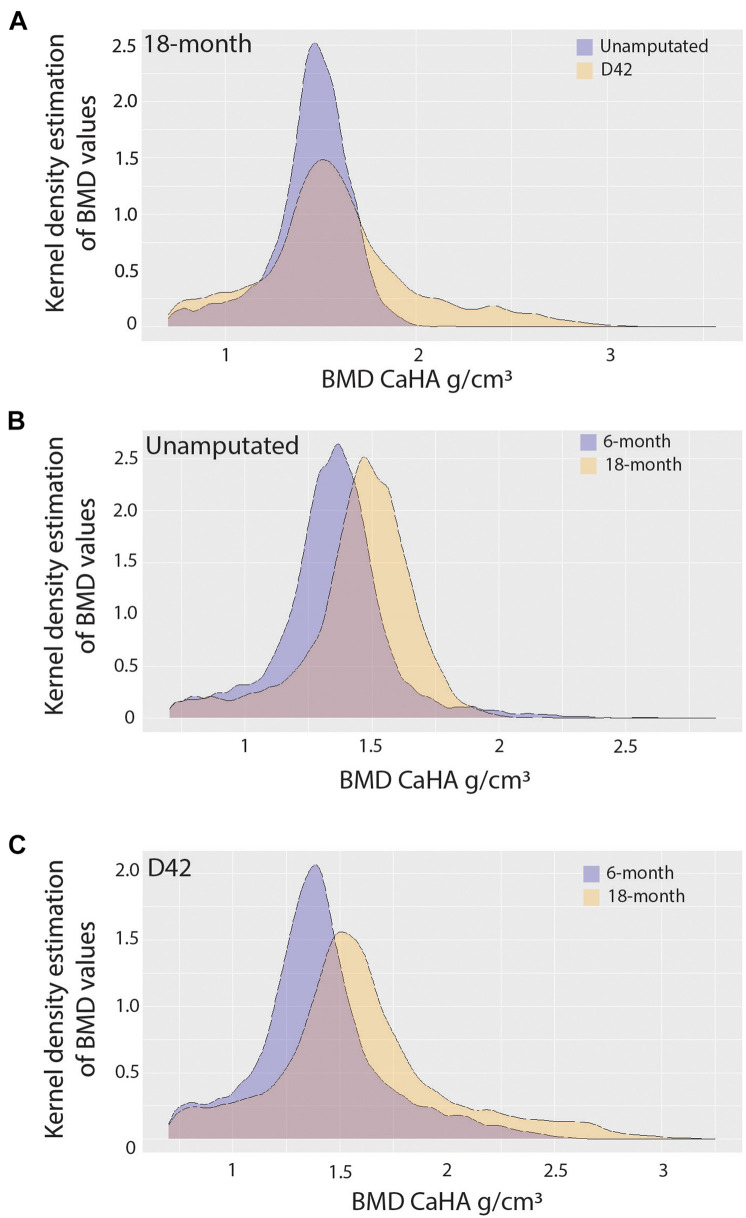
Bone mineralization. Pooled kernel density estimates compare **(A)** 18-month-old unamputated and regenerated digits, **(B)** 6-month-old and 18-month-old unamputated digits, and **(C)** 6-month-old and 18-month-old regenerated digits, showing the probability (frequency), and spread (density value range) of density values. Best-fit curve with normal distribution (significantly different *p* < 0.001). *N* = 10–12 digits.

**FIGURE 3:**
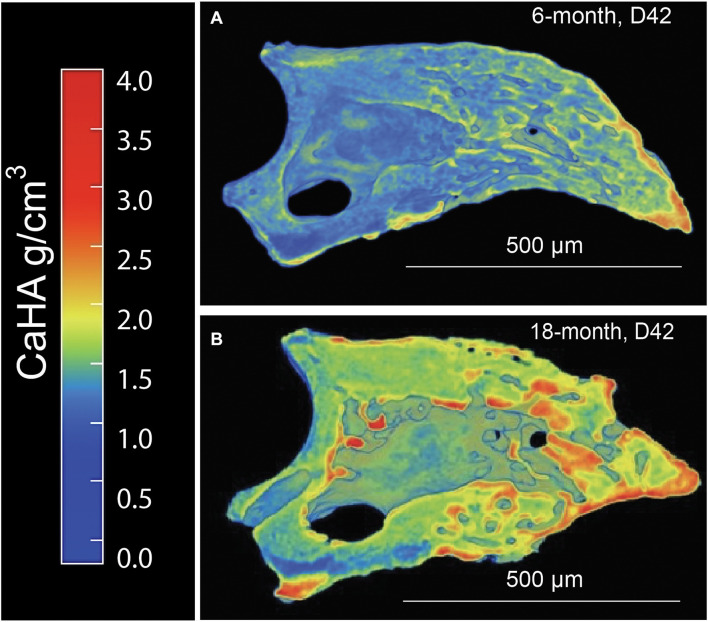
Distribution of calcium hydroxyapatite (CaHA) in microCT cross-sections of a **(A)** 6-month and **(B)** 18-month regenerated bone (D42). *N* = 11–14 digits. A representative sample is shown.

Since it is known that disease states such as osteoporosis decrease mineral density in an age-dependent manner compromising bone mechanics, we next sought to evaluate the impact of aging on mineral density in the regenerated aged digit. Mineral density analysis on aged digits using Python scripting (Hoffseth K. F. et al., 2021) showed that amputated digits had a significantly lower number of low-density values (10th percentile) than the D42 digit, a slightly higher median value, and a larger number of high-density values (0.75 and 0.9 quantiles; Figure 2A), similar to young regenerated digits (Hoffseth K. F. et al., 2021). Having previously analyzed young regenerated digits (Hoffseth K. F. et al., 2021), we then used this data to compare our aged digits with the young digits. Compared with the unamputated young digits, the unamputated aged digits showed an overall shift toward higher values of BMD distribution with significantly higher values (*p* < 0.05) for almost all modeled percentiles (10th, 25th, 50th, and 75th percentiles; Figure 2B). This age-dependent increase in mineral density was also seen in the regenerated digits where the aged mice also showed an overall shift toward higher values of BMD distribution, with significantly higher values (*p* < 0.05) in the same percentiles (Figure 2C). These higher mineralization values are predominantly localized in the distal regenerated bone and more specifically in the more central areas of trabecular bone. Interestingly, this increase in mineral density after an injury is not restricted to the regenerated bone and extends to the proximal bone stump as well (Figure 3). Together, these data show that the regenerative process increases the average and maximal mineral density levels and that aging exacerbates this effect. Furthermore, these changes in mineral density and mineral density distribution affect both the original bone stump and the newly regenerated bone.

### Nanoindentation

To better understand the effects of aging on the mechanics of skeletal regeneration, we employed nanoindentation to evaluate the impact of aging on the biomechanics of digit microstructure. After converting nanoindentation-derived reduced modulus values to Young’s modulus values (as shown in section “Materials and Methods”), we evaluated Young’s modulus and hardness measurements in both the proximal bone stump and the distal tip of unamputated digits and regenerated digits at day 42 in aged (18 months) mice. We compared the biomechanics of the sample microstructure at a regional tissue level (bone stump vs. regenerated bone). In unamputated digits, aged mice showed no significant change in hardness (*p* = 0.65) between the proximal and distal regions and no significant difference in Young’s modulus values (*p* = 0.82). Regenerated digits showed a significant decrease in both hardness (*p* = 0.001, 95% CI = 0.101 ± 0.066) and Young’s modulus in the distal regenerated bone compared with the uninjured original distal bone (*p* < 0.001, 95% CI = 1.96 ± 1.31). We then compared hardness and Young’s modulus measurements in our aged mice with 6-month-old young mice (Hoffseth K. et al., 2021). Interestingly, aged digits showed no significant difference in hardness or Young’s modulus between young and aged mice in all comparisons (UA, D42, distal/proximal; *p* > 0.05).

**FIGURE 4:**
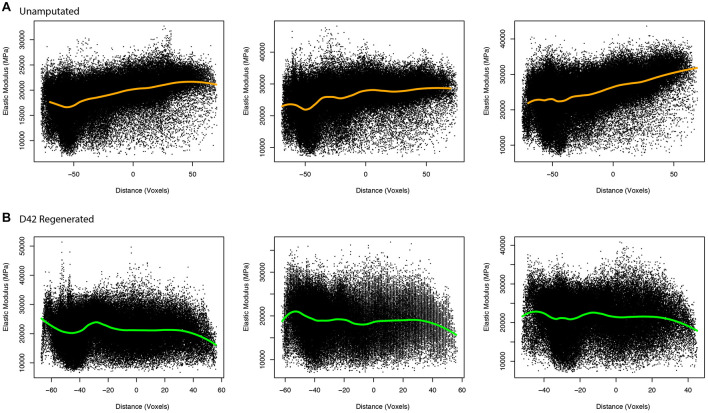
Elasticity in unamputated and regenerated aged bone. Proximal/distal plotting of numerically calculated **(A)** unamputated and **(B)** regenerated (D42) elastic modulus values of individual digits (*N* = 3 per group). The local polynomial regression curve indicated in green indicates the spatial trend in modulus value.

To better evaluate the aged regenerated bone and potential differences between aged and young bone, we utilized a modeling approach that allowed for numerical calculation of elastic modulus (Hoffseth K. et al., 2021). This approach uses μCT measured values of BMD to calculate predicted elastic modulus values for every representative voxel data point in the reconstructed digit, allowing for better predictive comparisons of the two groups. These comparisons of calculated elasticity were able to better detect differences within aged digits and between the two groups. The aged unamputated digit shows increased elasticity in the distal bone (*p* < 0.001, 95% CI = 4.6735, −4.1041), while the regenerated digit shows a significant decrease in elasticity distally (*p* < 0.001, 95% CI = 0.1736, 0.7430; Figure 4).

Collective comparisons of elasticity between the aged and young (Hoffseth K. et al., 2021) groups show that while both the proximal and distal areas of the unamputated digit are lower in elasticity in young mice (*p* = 0.004, 95% CI = −3.6464, −0.6651, and *p* < 0.001, 95% CI = −4.3190, −1.3377, respectively), the proximal and distal areas of the regenerated digits are not significantly different between the two age groups (*p* > 0.05). These data support that while the biomechanical properties of unamputated bone differ between age groups on a nanoscale, the regenerated bone in young mice is remarkably similar to the regenerated bone in aged mice with regard to elasticity.

## Discussion

To date, the murine model of skeletal limb regeneration has been analyzed at a tissue level using predominantly bone morphometrics. The goal of this study was to evaluate the impact of aging on regenerated bone, as well as evaluate the resulting biomechanics of the bone after regeneration. While the digit provides an excellent model for assessing skeletal regeneration, constraints involving the size and shape of the bone have made the biomechanical assessment of the P3 bone difficult. Nanoindentation allowed us to evaluate the mechanical properties of nanometer areas of bone tissue and gain further insight as to the attributes of regenerated bone. We previously developed and validated an image-processing method that allowed us to predict voxel elasticity measurements from density measurements in a 3D microCT stack (Hoffseth K. et al., 2021). This predictive method expands the elasticity analysis to the entire microCT stack (Figure 4), increasing the resolution of the elasticity analysis and consequently providing a more rigorous approach that moves beyond physical nanoindentation and individual measures of reduced modulus. Assessing the regenerative biomechanical outcomes of P3 as a model is important in determining the overall effects of altered gene expression and also treatments designed to promote regenerative outcomes. Curiously, what we found was that while elasticity and hardness were reduced in both aged and young (Hoffseth K. et al., 2021) distal regenerated bone compared with the original unamputated bone, there was no statistical difference between the two age groups, suggesting that the major age-dependent changes in skeletal regeneration are architectural.

Our data showing that both hardness and elasticity are reduced in regenerated bone are consistent with the findings in bone healing: newly formed bone has both reduced hardness and Young’s modulus when compared with the original cortical bone (Ishimoto et al., 2011; Vayron et al., 2011, 2012). Studies investigating the elastic moduli in bone lamellae from human cadaver samples using direct measurements also parallel our direct measurement nanoindentation data showing that Young’s modulus and hardness measurements demonstrated no correlation with age, albeit in uninjured bone (Hoffler et al., 2000). Taken together, our findings, combined with others, demonstrate that the aged skeletal regeneration model is able to produce a bone matrix that is similar in biomechanical properties to the young mouse, shifting our focus to age-dependent architectural differences.

Bone mineral density, on the other hand, was affected by both regeneration and age. Analysis of BMD showed that both regeneration and increased age resulted in BMD values that were skewed into the higher values. Prior data in aged sheep show that the normal process of aging increases mineralization in the trabeculae and subsequently increases mineralization heterogeneity (Brennan et al., 2014), similar to what we see during the regeneration and aging process. While this increase in mineralization is not enough to also increase the overall elasticity properties of the terminal phalange, increased BMD should also increase rigidity locally in those areas. This increase in BMD heterogeneity has been studied in other bone models, predominantly fracture healing. Bone remodeling (Frost, 1983, 1989; Ingle et al., 1999a) and BMD (Ingle et al., 1999b) are known to increase following fracture injury in humans. This localized response is accompanied by an overall decrease in BMD systemically, in order to increase the availability of minerals stored throughout the body (Osipov et al., 2018; Emami et al., 2019). Loss of heterogeneity in BMD has been attributed to increased risk of fracture, and drastically increased heterogeneity can concentrate strain in areas of low modulus and promote cracks (Osterhoff et al., 2016). Mechanical properties such as elasticity and hardness have been shown not to differ between healthy and osteoporotic bone (Nyman et al., 2016), due in part to the heterogeneity of the bone. While digit regeneration and fracture healing form bone *via* different processes (direct and endochondral ossification, respectively), the advantages of heterogeneity in BMD and elasticity and hardness in the digit model may parallel those seen in fracture healing, and further investigation should be focused on how this affects the regenerated bone.

Together our data suggest that aged mice are able to regenerate bone with the same elasticity and hardness of the bone observed in young mice. This pivots our focus to the spatial patterning of BMD in the regenerated bone and also to the bone architecture, both of which are altered in the regenerated bone of the aged mouse. Further work addressing the combination of both architecture and biomechanical properties, such as finite element modeling, will be extremely useful to further elucidate the quality of regenerated bone overall. In the meantime, our data are encouraging in those pathways that alter bone architecture and patterning can be targeted to promote better-regenerated bone, particularly in aged models, and suggest that methods to manipulate bone architecture in order to promote strength could be immensely helpful during regeneration. Identifying pathways that promote bone architecture changes, that promote better quality bone formation, and that increase bone mineralization will help to improve the process of regeneration. This analysis method will be impactful in evaluating the regenerative potential of treatments by addressing, at least in part, the biomechanical properties of the regenerated bone.

## Data Availability Statement

The raw data supporting the conclusions of this article will be made available by the authors, without undue reservation.

## Ethics Statement

The animal study was reviewed and approved by Institutional Animal Care and Use Committee of Tulane University Health Sciences Center.

## Author Contributions

MS, KH, EB, and JJ contributed to the data acquisition. MS, KH, EB, JJ, ML, and JS contributed to the analysis and interpretation of data. ML contributed to the statistical analysis. KH and MS contributed to the conception and design of the study, contributed to the writing and original draft preparation. All the authors have reviewed and agreed upon the last version of the manuscript.

## Conflict of Interest

The authors declare that the research was conducted in the absence of any commercial or financial relationships that could be construed as a potential conflict of interest.

## Publisher’s Note

All claims expressed in this article are solely those of the authors and do not necessarily represent those of their affiliated organizations, or those of the publisher, the editors and the reviewers. Any product that may be evaluated in this article, or claim that may be made by its manufacturer, is not guaranteed or endorsed by the publisher.
